# Faecal Attraction: 40 Years of Research in Gut Microbiology

**DOI:** 10.1111/nbu.70052

**Published:** 2026-04-22

**Authors:** Glenn R. Gibson

**Affiliations:** ^1^ Emeritus Professor, Department of Food and Nutritional Sciences The University of Reading Reading UK

**Keywords:** gut microbes, *Helicobacter pylori*, prebiotics, sulphate‐reducing bacteria

## Abstract

This article summarises the 2025 British Nutrition Foundation Annual Lecture given on 25/11/25. It overviews aspects of research in anaerobic microbiology, principally involving the human gut. Until October 2025, Gibson was Professor of Food Microbiology at the University of Reading. His research investigated gut microbiome interactions and dietary intervention. The latter included human studies in healthy persons, patients with gut‐related disorders, at‐risk populations and specialist groups like sportspersons and those in the military. Initial principles such as the type of intervention, dose, duration and outcomes were tested using in vitro models of the human gut.

## 1 Introduction

The human gut microbiome is the mixed community of microorganisms, including genetic components, microbial biodiversity and their resulting functionality, that inhabit the gastrointestinal tract. It is largely acquired at birth and colonises various organs to different extents. This is related to the availability of growth substrates which are largely provided by the diet, as well as other physicochemical aspects like transit time, pH and redox potential. As such, the highly acidic stomach harbours few microorganisms, while populations increase in the small intestine and then the colon where pH becomes more favourable and transit time gradually slows.

We live in good harmony with our microbiome and it is seen as essential for homeostasis, digestion, energy and immune modulation. However, this relationship can be compromised by poor diet, age, stress, lifestyle and antimicrobial intake. In fact, gut diseases are ubiquitous, being said to affect around 40% of the population of the world at any one time (Sperber et al. 2020). These range from acute issues like gastroenteritis through to chronic conditions such as Irritable Bowel Syndrome, Inflammatory Bowel Disease (Crohn's disease, Ulcerative Colitis (UC) and Pouchitis) and Digestive Cancers. Moreover, growing research is also investigating links between the microbiome and systemic conditions including atopic disorders such as asthma and eczema; cardiometabolic states and gut‐brain interactions. Evidence also points to a role for the gut microbial composition in responses to the SARS‐CoV‐2 infection, where gut health has been shown to have a role in symptomology and severity, including the development of long Covid (Lau et al. 2025). To varying extents, these conditions have all been linked to perturbation of gut microbial communities (Nagao‐Kitamoto et al. 2016; Chang and Lin 2016; Wang et al. 2024; Kraimi et al. 2024).

As the gut microbiome is seen to be pivotal for human health and has a large reliance on substrates provided by the diet, there has been much interest in the use of food‐based interventions to fortify beneficial components of the microbiome. This is the basis of the ‘functional foods’ concept whereby dietary ingredients are used for purposes over and above their normal nutritional value. Simply put, the gut microbiome and its activities can be modulated, opening up the possibility of addressing a range of healthcare and economic difficulties.

This has been the focus of my research for the last 40 years but had its origins with a PhD on a different microbial anaerobic environment. Here, various aspects of this are summarised. It will be evident that these experiences owe more to luck (good and bad) rather than intellect!

*As an aside, the first letter of the title from each section including this introduction, form an anagram of what has always been my favourite genus of bacteria*.

## 2 Estuarine and Marine Sediments

My PhD was carried out at the University of Dundee and Scottish Marine Biological Association, Oban (now Scottish Association for Marine Science). It compared the bacteriology of estuarine sediments (River Tay) with those at the bottom of marine lochs (Etive and Eil). The former involved core‐based sampling at low tide whilst the latter used drill borers launched from research vessels down throughout the water column, which could reach depths of up to 150m. These samples were then microbiologically examined, with particular attention being given to dissimilatory sulphate‐reducing bacteria (SRB). These are a group of microbes that use sulphate, or any sulphur‐based compound, as electron acceptors in respiration reducing this to hydrogen sulphide. The electron acceptor is organic material (and hydrogen), in these cases provided by sewage or industrial effluent. They are therefore especially important for optimising electron flow in anaerobic ecosystems, thereby contributing to energy gain for the microbiota. SRB are prevalent in marine or brackish sediments as seawater is plentiful in sulphate. On the contrary, freshwater sediments where sulphate is depleted tend to have methanogenesis as an alternative. Hydrogen sulphide is of course very noxious (think rotten eggs) but reacts well with metals. In the case of iron, this forms ferrous sulphide which can give sediments a characteristic black colour. It is also problematic in terms of compromising the integrity of metal‐based pipelines and tanks through corrosion, but importantly is toxic to humans at high enough levels.

## 3 Obligate Anaerobes in the Gut and Interspecies Hydrogen Transfer

Given the potential pathogenicity of SRB, my interest was then in whether they could have effects in other anaerobic ecosystems, the obvious choice being the human gut. My research at MRC Dunn Clinical Nutrition Centre in the 1990s looked at the intestinal microbiome of humans and the role in health. The ‘gut group’ was one of the few groups carrying out such research, led by John Cummings who pioneered research into the effects of fibre fermentation. The other microbiologist was George Macfarlane, my sadly deceased friend and colleague. Together we investigated hydrogen uptake following microbial breakdown of dietary ingredients. Like in sediments, hydrogen is readily produced by microbes but needs to be disposed of to allow better energy flow. From the human gut, we isolated many strains of SRB belonging to different genera. It became apparent that they were active only in around 50% of the population, the other tending to generate methane by combining H_2_ with CO_2_. Key to this was diet, with persons on highly sulphated diets tending towards SRB as the main hydrogen disposal route (Gibson, Cummings, et al. 1988; Gibson, Macfarlane, et al. 1988).

From there, the research then investigated the pathology of SRB growth, particularly the formation of H_2_S. We looked at the debilitating inflammatory bowel disease UC. UC is a severe chronic inflammatory disease confined to human large intestine, which affects about 10 people in every 100, 000 in Western populations. Onset most frequently occurs between the ages of 20 and 35 years. Symptoms are typically bloody diarrhoea and occasionally abdominal pain. In up to 30% of cases, surgery is required. We found that SRB were far more common in the UC gut, could generate much higher quantities of toxic sulphides than equivalent strains from the ‘healthy’ colon and were able to adapt to certain clinical manifestations of the disease such as a rapid transit of gut contents and low substrate availability (Gibson et al. 1991).

## 4 Very Fastidious Microorganisms

As mentioned by Postgate (1984) in his seminal work, SRB are tricky to grow. They do not do well on petri dishes and instead are cultivated using complex media, in so‐called agar shakes. These are test or Hungate tubes filled with semi‐solid agar. SRB grow as black coloured colonies when iron is present in the medium. However, they cannot compete for fastidiousness when compared to *Helicobacter pylori* (another microorganism that grows very well in humans but poorly in laboratories).

*H. pylori* is a notorious stomach pathogen notable for its links to peptic ulcer, Type B gastritis and gastric carcinomas. In the 1990's, treatment for *H. pylori* carriage was limited to use of antimicrobials which is still the case today albeit with a wider range and other pharmaceuticals in conjunction. It was apparent that following successful treatment, *H. pylori* could frequently re‐infect the patient even after stomach clearance had still been confirmed. This suggested that the pathogen was present in other gut areas, with the large intestine being obvious. We therefore attempted to isolate *H. pylori* from faeces. At the time (early 1990's), today's sophisticated molecular methods for characterising the gut microbiome were not advanced. As such, our efforts were based around cultivation on blood containing semi‐selective media (Skirrow, Dent). This systemically failed time after time. However, this ‘failure’ caused us to think a little more laterally and we had the idea on centrifuging faeces to make a concentrated pellet of bacteria. Should *H. pylori* be present, this would give a far higher mass for inoculation on the agars. This straightforward trick in fact resulted in *H. pylori* recovery and was the first of its isolation from faeces. The research confirmed that colonic carriage of *H. pylori* was feasible and that future treatments and detection ought to take this into account. To this day, I am still surprised that such an easy thought overcame the initial issue. Indeed, we were able to publish in The Lancet—which would not have been possible had the original isolations worked out (Thomas et al. 1992; Kelly et al. 1994).

## 5 Using Gut Models

Prior to carrying out human intervention studies on the gut microbiome, initial assessments can be made in vitro using ‘gut models’. Ours were first developed in Cambridge and ranged from simple static anaerobic batch culture fermenters to multiple stage continuous cultures. The latter replicates different anatomical areas of the colon. It was originally validated against gut contents from sudden death victims and gives analogy to bacterial activities and composition in different areas of the hindgut (Macfarlane et al. 1998). The system consists of 3 vessels, of increasing size, aligned in series such that sequential feeding of growth medium occurs. The vessels are pH regulated to reflect in vivo differences. Thus, vessel 1 has a high availability of substrate, bacteria grow quickly and is operated at an acidic pH, similar to events in the proximal colon. In contrast, the final vessel resembles the neutral pH, slow bacterial rate and low substrate availability which is characteristic of more distal regions. After inoculation with faeces, an equilibration period is allowed such that the bacterial profiles respond to their imposed conditions, the continuous flow of medium eventually allowing a steady state condition. Test ingredients (e.g., probiotics, prebiotics, other bioactives, diets, pharmaceuticals) are then added and a further steady state condition reached. Studies on development of the microbiota in the three vessels are carried out including measurements of composition and activity. The advantage of such a model is that it can predict microbial events in different areas of the colon, thereby leading to fundamental information on mechanisms of effect. These approaches can be used to identify which test can then be performed in vivo including dose, duration.

## 6 Beneficial Microbes

As the gut microbiome is seen to be pivotal for human health and has a reliance on substrates provided by the diet, there has been interest in the use of food‐based interventions to fortify beneficial components. Traditionally, this has been through the use of probiotics as live microbial feed supplements (Hill et al. 2014). These have a long history of use in humans and have been the subject of over 50, 000 research papers. Probiotics must be safe, amenable to industrial processes necessary for commercial production, remain viable in the food product and during storage and, importantly, persist in the gastrointestinal tract long enough to elicit effects that can improve host health. Because of their positive properties and safety, lactic acid producing bacteria have been the usual constituents in probiotics. These predominantly include lactobacilli and bifidobacteria. Other probiotics have included yeasts, *Bacillus* spp. and certain Gram‐positive cocci.

Arguably, this is a narrow viewpoint of the health promoting properties of the gut microbiota that does not exploit the full potential. For example, butyrate is often suggested as the most desirable of all gut metabolites because it is a good oxidisable fuel for colonocytes. However, most current probiotics do not manufacture this metabolite. The same applies to propionate, which is touted as an appetite regulator. Other aspects possibly that can enhance the probiotic repertoire include anti‐inflammatory functions, the ability to reduce gas distension issues including ‘greenhouse’ emissions, anti‐viral aspects, reduced tumorigenesis. As such, as our knowledge of gut microbial diversity continues to expand into other possible health promoting genera that may offer opportunities beyond those of traditional probiotics, for example, *Roseburia* spp., *Faecalibacterium* spp., *Eubacterium* spp., *Akkermansia* spp., *Christensenella* spp. and *Propionibacterium* spp. Time will tell whether these become common as probiotics with considerations such as safety, manufacture and in vivo potential to be confirmed.

## 7 First Human Intervention Study

My first PhD student was Xin Wang. She was on an industrially funded studentship to look at the effects of chicory root derived inulin (a fructooligosaccharide) on the colonic microbiota. Specifically, whether its ingestion could boost beneficial bifidobacteria. The theory being that the microbes could metabolise β‐fructanoside bonds in the molecule, owing to appropriate cell‐associated enzymes and grow selectively. Following a series of in vitro assessments from pure to mixed culture, we carried a human intervention study (Gibson et al. 1995). This involved the grand total of eight volunteers only, however there was a good reason for that very low number. In those days, gut microbiology was carried out using conventional culture techniques. It was unheard of that molecular biology would ultimately provide tools that could characterise the entire microbiome without inoculating any petri dishes and even encompass species unable to grow in vitro. We used various purportedly selective growth media designed to cover major gut genera known at the time. Of course, these exploit antibiotics or similar agents to impart selectivity but that is never wholly the case, that is, a growth medium designed for bifidobacteria will recover related groups too. As such, Xin painstakingly used biochemical and staining procedures to characterise every colony which grew—on hundreds and hundreds of petri dishes. Inulin had the anticipated effect but only in the low study number we used. Nevertheless, this study was pivotal to our developing the concept of prebiotics.

## 8 Defining Prebiotics

As part of Xin's research, I was introduced to Marcel Roberfroid, Universite Catholique de Louvain, Belgium. We would have frequent research meetings when he visited London. These were in the Scandic Crown Hotel, Victoria. At one of these, we considered that inulin acted like a probiotic in that it was selectively changing the gut bacteriology positively, but as it was inert did not have possible survival issues in the product or gut. We decided to write a review on the research and other similar studies showing how carbohydrates could selectively fortify beneficial gut bacteria like bifidobacteria. I suggested we should give this concept a name and we agreed to think about possibilities. I went home and started drafting this review. A few hours later that was sent to Marcel who then turned my words into something more like science.

Then, we needed to give the concept a name and include that in the article title. I favoured ‘parabiotics’. At the time, M*A*S*H* (Mobile Army Surgical Hospital) was a popular comedy programme in the UK. It was an American series set in the 1950's Korean War. In it, paramedics would feature and these were people who helped medics. My thinking therefore, was that a parabiotic would be something to help biotics. But in the end, we decided on prebiotics (not to be confused with primordial soup). We went with that and called the paper ‘Dietary Modulation of the Human Colonic Microbiota – Introducing the Concept of Prebiotics’. It was published in the Journal of Nutrition (Gibson and Roberfroid 1995). Little did we know the impact that would follow for a review that took only a few hours to write would have, with prebiotics now being the subject of over 18 000 research articles, the subject of many conferences, countless products, new companies and even a research organisation (see later). The irony is that the original paper is too simplistic, massively outdated now and the definitions in there (we also defined synbiotics for the first time) are far too wordy. To verify this, the Journal of Nutrition rejected an update of ours 10 years later!

## 9 Other Human Studies

My research since moving to Institute of Food Research, Reading (in 1995), then University of Reading (in 1999) moved away from microbial pathogenesis towards prebiotics. Table 1 shows a list of human studies carried out, to date, in this regard. Of course, some were successful, others moderately so, whilst several showed nothing. One fascinating aspect has been to see how characterisation of the gut microbiome has evolved following the ‘molecular revolution’ in the late 1990s. Moreover, other techniques such as metabonomics have vastly improved the studies as have valid biomarkers of health and clinical symptomology.

**TABLE 1:** Examples of prebiotic human intervention studies.

Study population	Intervention carried out
Healthy persons	FOS containing biscuits Fermented dairy drinks Blackcurrant juice GOS AXOS containing bread Confectionery sweeteners Traveller's diarrhoea Polydextrose XOS MOS GlOS Fruit shots containing FOS Inulin from globe artichoke, agave, chicory Rugby players USA military High protein diets Herbs Breakfast cereals (oats, rice, whole grain wheat) Bran products Snack bars Sports drinks Cocoa‐derived flavanols Orange juice Yoghurt
Patients	Ulcerative Colitis Irritable Bowel Syndrome Colorectal cancer Peptic ulcers Cardiometabolic health Autism Diabetes Antibiotic Associated Diarrhoea Stress Enteral feeding
Age related populations	Infants Elderly

Abbreviations: AXOS, arabinoxylooligosaccharides; FOS, fructooligosaccharides; GlOS, glucooligosaccharides; GOS, galactooligosaccharides; MOS, mannooligosaccharides; XOS, xylooligosaccharides.

At this stage, I must acknowledge the dedicated efforts of all the wonderful PhD students, postdoctoral researchers, technicians, and other colleagues, including research sponsors, who did all the hard work. Ongoing studies in gut‐brain axis, seaweeds, artisan cheeses, and teas are occurring.

## 10 Synbiotics

A further concept for altering the gut microbiota for improved health exists. This is known as synbiotics and exploits both the above approaches—therefore having the capacity to be more functionally efficacious. Simply put, a synbiotic is a mixture of a reliable probiotic with a prebiotic. The word synbiotic is derived from the Greek ‘συν’ and ‘βίος’ which translates as together and life—signifying synergy in the Greek meaning.

International Scientific Association for Probiotics and Prebiotics (ISAPP, see later) has defined them as ‘mixtures comprising live microorganisms and substrate(s) selectively utilised by host microorganisms that confer a health benefit on the host’ (Swanson et al. 2020). Hence, one approach would be to fortify strain activity (probiotic) by incorporating a selective substrate for growth (prebiotic) within the competitive gut ecosystem. This would be a synergistic synbiotic, while ISAPP also proposed complementary synbiotics where the individual entities act independently.

In either case, the advantages are that a probiotic with known benefits can be used and the prebiotic should further promote establishment of live microorganism(s) in the complex gut environment. A good synbiotic would exploit positive properties of both the probiotic and prebiotic ingredients—whether together or separately. As such, expected benefits of synbiotics (depending on whether they are synergistic or complementary) could be as follows:

- Improved survival during passage of the probiotic(s) through the upper gastrointestinal tract (synergistic).
- A more persistent effect of the probiotic(s) within the host gut ecosystem—bearing in mind that most gut diseases have a distal origin (synergistic).
- The prebiotic may stimulate indigenous components of the colonic microbiota considered to be beneficial (complementary).
- Health advantages from the synbiotic may ensue from each individual ingredient (both synergistic and complementary).

It is possible therefore that synbiotics offer more flexibility and efficiency than probiotics or prebiotics alone. However, they have not attracted anywhere near the amount of scientific nor product developmental interest that the other approaches have.

## 11 Linking Research With Sports and Travel

Gut microbiology research has been very good to me, even allowing the privilege to travel to most countries that I have ever wanted to visit and making friends all over the world. As an aside, it was quite a surprise, on a flight to Chicago, to see our research featured in an advertisement in British Airways HighLife magazine. This was in relation to repressing gastroenteritis while on holiday and came from our publication in the European Journal of Nutrition (Drakoularakou et al. 2010) which investigated the effects of a prebiotic on Traveller's Diarrhoea. I have always felt that the scientific rigour and refereeing standards of HighLife rival those of Nature, Science, PNAS, etc.!

The same publication also came to the attention of sports groups—allowing me to combine research with the other interest that has always dominated my life. The product we tested in this study was used by Team GB Olympians, Commonwealth Games athletes and the GB side in World University Games. It then was tested in a professional rugby union team—happily one that I followed for decades (Parker et al. 2023). Only Sunderland football and Durham cricket clubs to go!

## 12 Reaching Out

**FIGURE 1:**
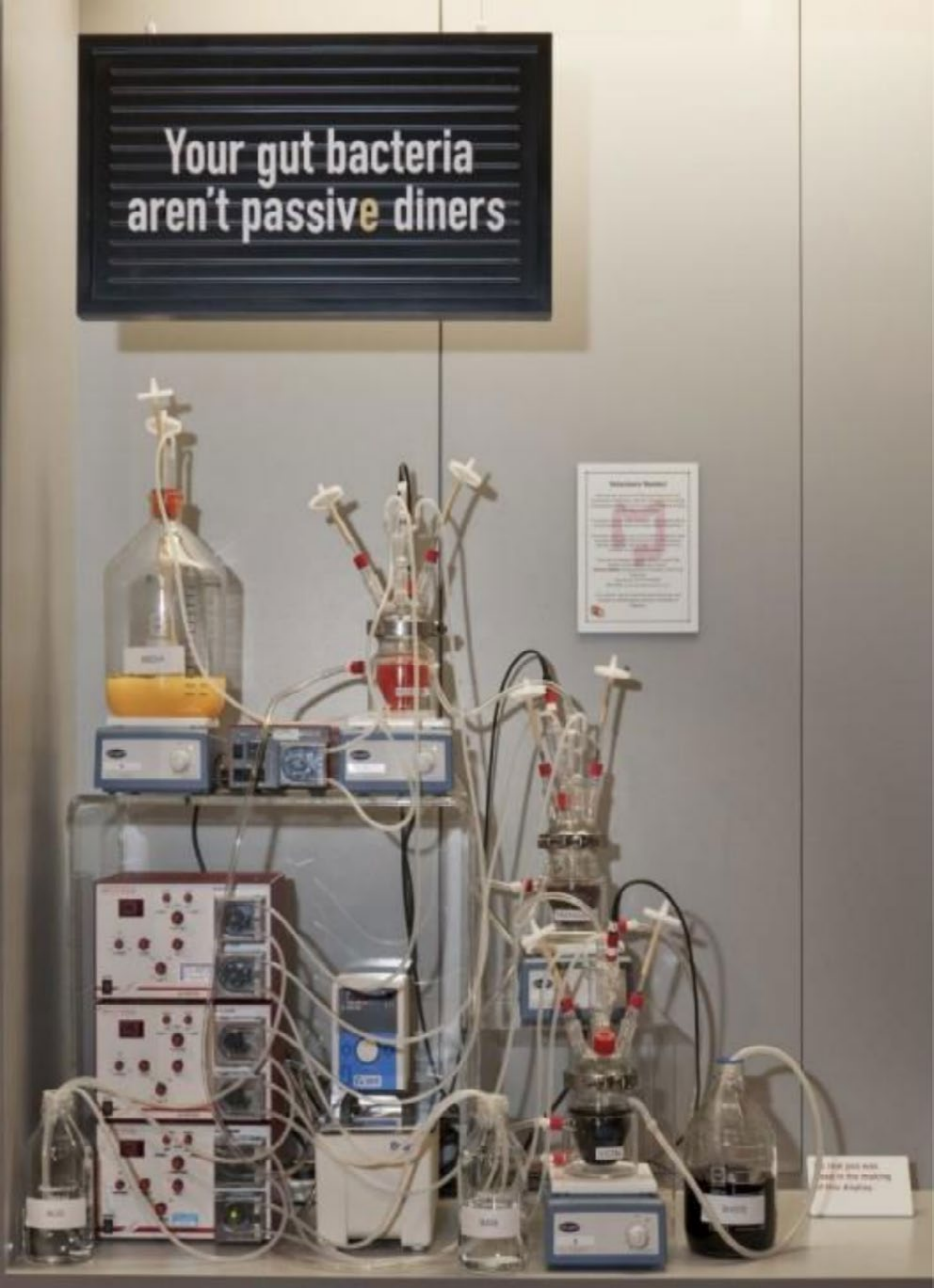
University of Reading ‘gut model’ on display at the London Science Museum as part of a demonstration on food cravings.

The gut microbiome field has surged in popularity since our early days in Cambridge when it was very much a niche science. Along with this, outreach opportunities have arisen. These have included traditional routes such as lay presentations, media interactions, board memberships, school/college visits, podcasts and blogs. However, three aspects spring to mind which I would never have expected to occur:

- *Royal Society Summer Show*: This exhibition is the Society's public event of the year open to member of the general public, students, teachers, scientists, policymakers and the media, typically attended by over 12, 000 visitors with coverage on TV, in the print media and online. In 2007, we put together a functional foods demonstration called ‘A Microbial Journey Through Digestion’. It looked at probiotic microbes involved in food production through to effects in the gut.
- *All Party Parliamentary Group (APPG)*: In 2019 my colleagues Drs Gemma Walton, Kirsty Hunter, leading campaigner Alan Barnard and myself helped set up an APPG on the gut microbiome. This was chaired by Julie Elliot MP. It included a group of MPs and peers that had an interest in the field. The aim was to increase knowledge among parliamentarians on the potential benefits of the gut microbiota and its modulation to improve health. Actions included: in person meetings and presentations; development of briefing documents for APPG meetings (e.g., probiotics and prebiotics; sports persons health, developing world, *Clostridium difficile* infections; Irritable Bowel Syndrome; gut to brain axis); working groups on particular items such as health claims; identification of specific constituency relevance; drafting house questions to relevant ministers, and hosting parliamentary visits to our laboratories.
- *Working with Museums*: This has included gut microbiology demonstrations at Universities Week (Natural History Museum, London) and BBSRC's Great British Bioscience Festival. The latter contributed to the ‘Colon Café’ exhibit housed at Winchester Science Museum. In 2015, and until April 2016, an example of our gut model was present in the Science Museum, London as part of an exhibition on cravings (Figure 1). In 2017, this then transferred to the Manchester Science and Industry Museum.

Aside from the great enjoyment the above brought, particular in terms of doing something different, these were effective ways of allowing our research to reach audiences that otherwise we would not have.

## 13 International Scientific Association for Probiotics and Prebiotics

The last word goes to ISAPP. This is a non‐profit organisation dedicated to probiotic and prebiotic (now also synbiotic, postbiotic) research. It provides a forum for researchers, food and pharma industries to collectively discuss pro and prebiotics. ISAPP produces fundamental research papers, training for early career researchers, media outputs, webinars, infographics, videos and consumer commentaries on the research area. It also awards 3 research prizes annually.

The idea of forming ISAPP was in 1999, when the boundlessly energetic probiotics expert Mary Ellen Sanders (Denver, Colorado) and myself were part of a conference organising committee. We discussed that, at this time, no specific ‘home’ (i.e., research organisation) existed for probiotics and prebiotics. Rather, these areas were adjuncts in other microbiology and nutrition societies. We decided to address this and gauge interest from a small group of other researchers. The idea gained much momentum when we engaged the equally energetic and productive Gregor Reid (London, Ontario). A kind financial donation from Danone (via the efforts of Irene Lenoir‐Wijnkoop) allowed us to host an inaugural meeting at Gregor's institute in London, Ontario and put together a Board of Directors. There has been an annual conference ever since.

Of my meagre academic interactions, being involved in the instigation of ISAPP is the proudest.

## 14 Anagram Letters

I

E

O

V

U

B

F

D

O

S

L

R

I

(You can email me if you want the solution).

## Funding

The author has nothing to report.

## Conflicts of Interest

The author declares no conflicts of interest.

## Data Availability

Data sharing not applicable to this article as no datasets were generated or analysed during the current study.
